# Feeding ecology of the endangered Asiatic wild dogs (*Cuon alpinus*) across tropical forests of the Central Indian Landscape

**DOI:** 10.1038/s41598-022-17906-5

**Published:** 2022-08-18

**Authors:** Pallavi Ghaskadbi, Neetu Bathla, Aishwarya Bhandari, Shrushti Modi, Parag Nigam, Bilal Habib

**Affiliations:** Wildlife Institute of India, Dehradun, India

**Keywords:** Ecology, Evolution, Zoology

## Abstract

Studies on resource utilisation by carnivores are essential as they aid in assessing their role in a community, by unravelling predator–prey relationships. Globally, prey depletion is one of the primary causes of declining Asiatic wild dog (dhole) populations. Therefore, it is essential to examine their diet across their range. Our study presents insights into dhole feeding ecology across multiple sites from the central Indian landscape of Maharashtra, India, for the first time. We conducted scat analysis using a subset of genetically identified scats and collected additional data from kills observed while tracking radio-collared dholes and other known packs from 2 study sites. We analysed 861 scats, and 191 dhole kills to identify species and age class of prey. We estimated the relative contribution of various prey, utilising non-linear biomass models of prey consumption. Overall, wild ungulates like sambar and chital were the principal prey in terms of biomass (sambar 61.08%; chital 19.08%) and number of prey consumed (sambar 39.28%; chital 13.83%). An analysis of kill data also suggested that dholes strongly preferred the two deer species; and differential selection of age classes was observed at the 2 study sites. Our study can potentially help manage and conserve this important population of an endangered carnivore.

## Introduction

Diet analyses by investigating faecal remains of an animal have been a fundamental part of carnivore ecology and natural history studies^1–4^. As carnivore populations are closely associated with prey density and biomass^5^, understanding ecosystem effects must begin with accurate knowledge of predator diets^6^. Findings from carnivore diet studies may have far-reaching impacts on management plans of endangered species in particular^4^ and even assist in structuring and implementing relevant conservation measures^7–9^. From an applied perspective, livestock depredation and perceived competition with humans have driven the global decline and local extinction of carnivore species^10,11^, emphasizing the need for rigorous identification of carnivore diets^6^. Additionally, an assessment of the dietary niche breadth may reflect geographical differences among populations^12^. Therefore, an understanding of the site-specific feeding habits is of high conservation value as it shapes population structure, and determines ecosystem interactions.

The Asiatic wild dog (*Cuon alpinus, Pallas 1811*) or dhole, listed as an endangered carnivore by the IUCN^13^, is an apex predator of the South and South-East Asian forests^14,15^. The dhole is the only social, forest-dwelling canid found in closed forests across its range in Asia, primarily restricted to protected areas^13,16^. However, threats such as habitat loss, fragmentation and human persecution have resulted in an estimated 82% decline in its original range^17^. India is home to the world's largest remaining dhole population^13^, but over the last century has lost approximately 60% of its original habitat^18,19^. Evidence suggests that depletion of their prey base has resulted in a range contraction of this species^13^.

Large canids are also known to cause trophic cascades when their populations fluctuate^20,21^. As apex predators evolved to prey predominantly on a carnivorous diet comprising ungulates, dholes potentially contribute to maintaining trophic interactions by influencing prey populations. Therefore, information on the foraging habits of an endangered hypercarnivore^22^ like the dhole from this previously unexplored landscape of high biodiversity significance and conservation value would be critical for their conservation and management^23^.

Traditionally, diet studies on dholes in India employed the scat analysis method and direct observations at kill sites to study diet preferences (See Supplementary Table S1). In the recent past, studies have reviewed dhole diets across their range with reference to competition within the co-predator guild^24^ and risks associated with human interactions^25^. Although historically, dholes were treated as vermin and persecuted across India^15,26^, the country remains home to the largest remaining dhole population^13^, limited primarily to 3 landscapes—the Western Ghats, Central India and the North East^25^. From a literature review of diet assessments (n = 22), we found that merely 18% (n = 4) of the studies were from the Central Indian landscape (henceforth CI). The CI landscape comprising of 6 states supports nearly 50% of the dhole occupied area^27^, however, there is an apparent paucity of studies on feeding habits of dholes from CI that is one of the strongholds of its population^28,29^. The imbalance is further highlighted at a finer scale wherein we found no diet studies from Maharashtra—a state ranking among the highest in the current status, recovery and survival of dholes and highest in conservation priority for the species^27^. As the State Forest Departments in the country are primarily accountable for wildlife and forest management, our study can guide conservation efforts for endangered species.

In this study, we aimed to gain insights into the dietary preferences of dholes across their metapopulations in the fragmented landscape of CI following an integerative approach. We conducted faecal examinations of dhole scats collected from 6 protected areas using a combination of opportunistic sampling and by following radio-collared dholes in the field. These samples were analysed utilizing the exponential biomass equation to study the dhole diet profile. An exponential relationship is biologically and physiologically more realistic than a linear function, as it predicts that the amount of prey consumed by a carnivore to excrete one scat reaches an asymptote at large prey sizes^30^. Further, we included direct kill observations from 2 of the field sites—Tadoba Andhari Tiger Reserve (TATR) & Nawegaon Nagzira Tiger Reserve (NNTR). The 2 specific objectives of the diet study were to:

1. Determine the frequency of occurrence of different prey items in the dhole diet, relative biomass, number of prey taken of principal prey species and niche breadth on a landscape and PA scale;
2. Determine the age classes of principal prey based on kills data obtained from tracking and observing dholes in the field.

## Results

### Scat analysis

Our overall sample size was large enough to draw inferences about the dietary habits of dholes across the landscape, given that the minimum sample size was 40 samples (See Supplementary Fig. S2). Although most scats contained a single prey type (59.47%), at times, multiple prey types were also found, resulting in the average prey item per scat value of 1.44 for dholes. About 76% of dhole scats also contained varying amounts of grass (mainly bamboo *Dendrocalamus strictus*) with or without prey remains. Table 1 provides an overview of the dietary results at the landscape scale.

**Table 1:** Average live weight of prey consumed (X), frequency of occurrence (A), weight of consumed prey represented by one field-collectible scat using correction factor (Y), relative biomass consumed (D), and relative number of prey individuals consumed (E) by dholes based on scats collected across the study sites in Maharashtra, India (n = 861).

Prey	X kg	A%	Y	D%	E%
Chital	55	15.55	0.95	19.08	13.83
Sambar	62	46.84	1.01	61.08	39.28
Barking Deer	20	4.34	0.47	2.66	5.31
Black naped hare	3	7.07	0.08	0.77	10.27
Gaur	75	2.83	1.10	4.02	2.14
Nilgai	70	3.39	1.06	4.68	2.67
Rodent	0.25	5.66	0.01	0.05	8.46
Chausinga	19	5.84	0.45	3.44	7.23
Langur	8	5.37	0.21	1.49	7.42
Wild pig	31	3.02	0.66	2.58	3.32
Cattle	75	0.09	1.10	0.13	0.07

The most frequently preyed upon species by dholes were sambar *Rusa unicolor* (46.84%) and chital *Axis axis* (15.55%), followed by black-naped hare *Lepus nigricollis*, rodents, chausinga *Tetracerus quadricornis* and langur *Semnopithecus entellus*. Consistent with the frequency of occurrence, the biomass model also suggested that sambar (61.08%) and chital (19.08%) were the principal prey of dholes across the landscape (Table 1).

**Figure 1:**
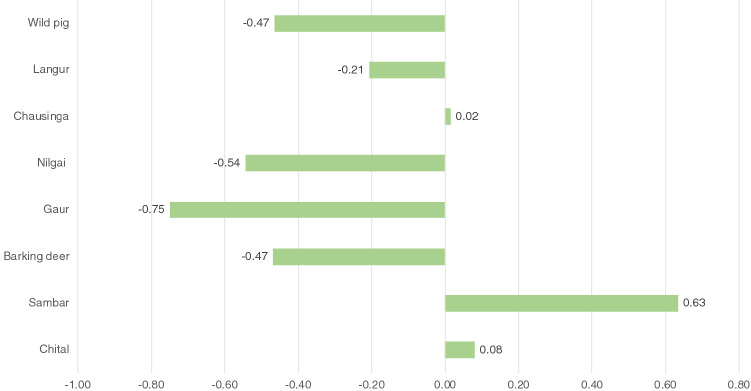
Jacobs’ index for prey preference in dhole diet across the central Indian landscape. The scale ranged from − 1 to + 1 representing strong avoidance and strong preference respectively. Sambar was the most preferred prey of dholes followed by chital and chausinga. Gaur, nilgai and wild pig were the least preferred prey.

We calculated Jacobs’ index scores of 9 species recorded as dhole prey. Based on the Jacobs’ index score, dholes significantly preferred to prey upon sambar (0.63) and chital (0.08) (Fig. 1). Along with these two ungulate species, chausinga (0.02) was found to be the preferred prey of dholes at the landscape scale. There was an evident avoidance of species like gaur *Bos gaurus* (− 0.75) and nilgai *Bosecephalus tragocamelus* (− 0.54) by dholes (Fig. 1).

**Figure 2:**
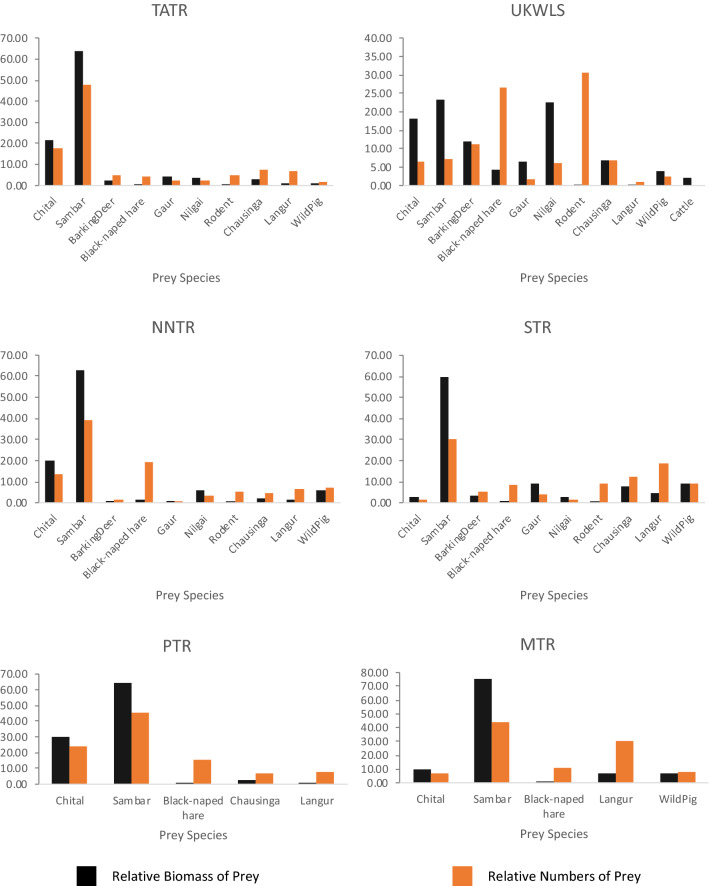
Relative biomass of prey species and relative numbers of prey harvested by dholes across the study sites, Maharashtra, India: *TATR* Tadoba Andhari Tiger Reserve, *NNTR* Nawegaon-Nagzira Tiger Reserve, *STR* Sahyadri Tiger Reserve, *UKWLS* Umred Karhandla Wildlife Sanctuary, *MTR* Melghat Tiger Reserve, *PTR* Pench Tiger Reserve.

In consistence with the landscape scale findings, sambar and chital were the prey species most commonly preyed upon by dholes at an individual PA scale as well (Fig. 2). Based on the cumulative values of the PA-scale analysis, sambar and chital alone contributed three quarters (75.11%) of the biomass of 11 prey species consumed by dholes. However, in Umred Karhandla Wildlife Sanctuary (UKWLS), nilgai was the second most important prey (22.50%) after sambar (23.20%), followed by chital (18.19%) based on the biomass consumed (See Supplementary Table S3). The data also indicated that the gaur is harvested in PAs like Sahyadri Tiger Reserve (STR) (9.0%), UKWLS (6.3%) and TATR (4.17%). Dholes invariably kill small-sized prey like black-naped hare and rodents if they are present at a site.

Our results from the niche breadth analysis of dholes indicate that scats sampled from UKWLS had the widest dietary niche breadth (Levins’ Index = 6.4; N = 84), followed by STR (Levins’ Index 4.8; N = 107), NNTR (Levins’ Index = 3.6; N = 118), TATR (Levins’ Index = 3.0; N = 67) and a narrow dietary niche at Melghat Tiger Reserve (MTR) (Levins’ Index = 2.8; N = 12), and Pench Tiger Reserve (PTR) (Levins’ Index = 2.8; N = 16) [but note the sample size < 40 for the latter two PAs.]

### Kills analysis

**Figure 3:**
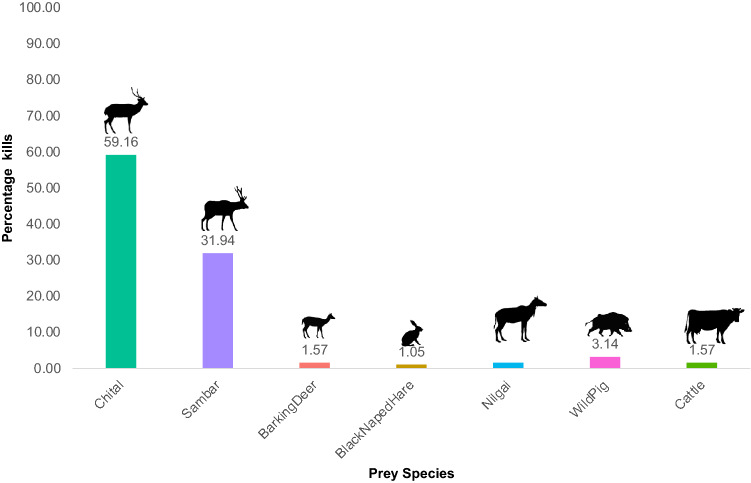
Proportion of prey species in dhole diet based on overall kill observations of all species in field from two study sites (TATR and NNTR).

We recorded 191 kills of various prey species from two study areas—TATR (160) and NNTR (31). Of the total kills, chital was the most commonly encountered prey (59.16%), followed by sambar (31.94%). We also recorded dholes feeding on wild pigs, nilgai, barking deer, cattle, and black-naped hare (Fig. 3).

**Figure 4:**
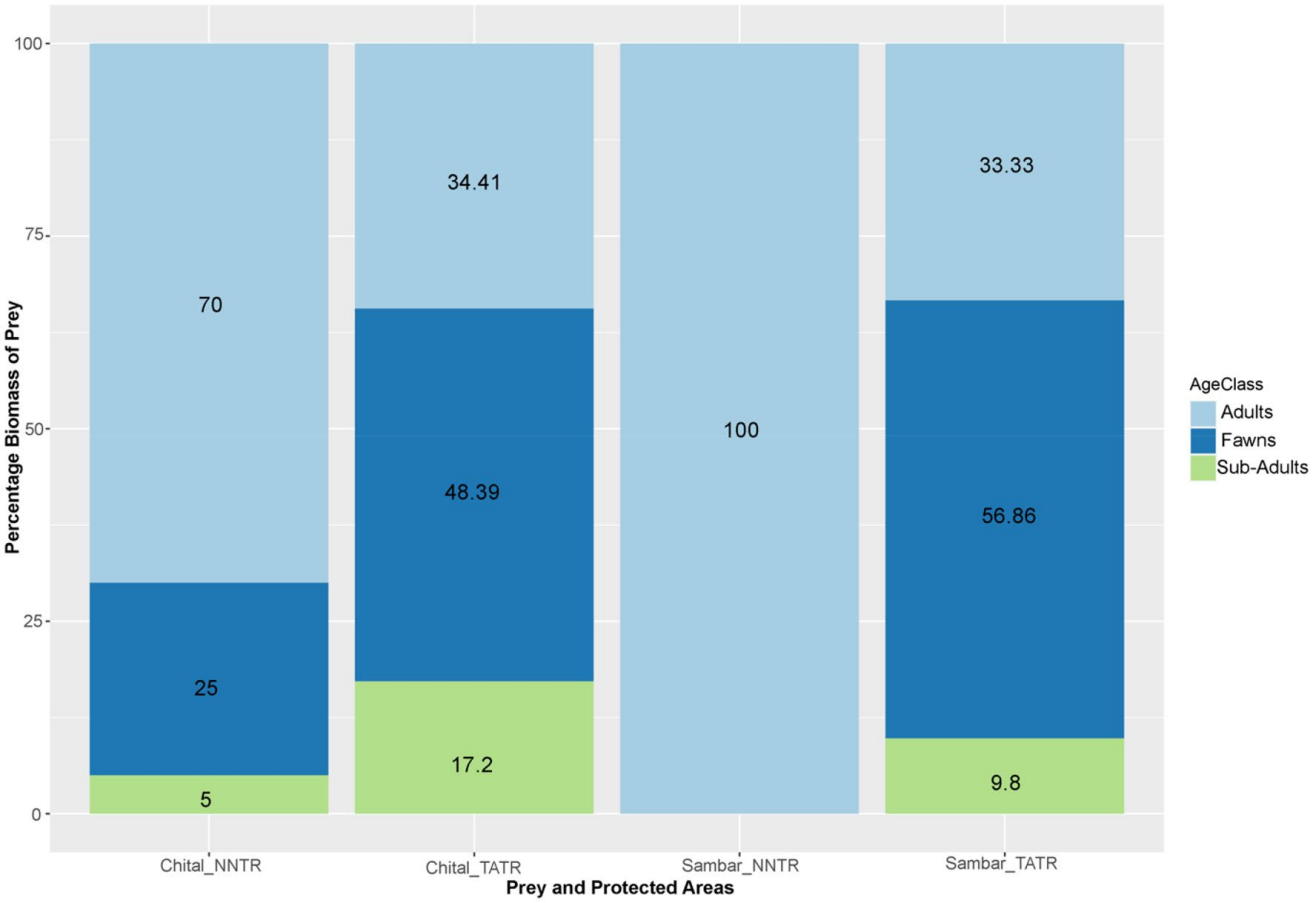
Proportion of age classes (Fawns, Sub-Adults and Adults) of chital and sambar found from the dhole kills in TATR and NNTR. Figure generated using the package “ggplot2” in R studio R Core Team (2019). R: A language and environment for statistical computing. R Foundation for Statistical Computing, Vienna, Austria. (https://www.R-project.org/).

The analysis of recorded kills demonstrated that in NNTR, chital and sambar adults contributed more to the diet composition of dholes (70% and 100%, respectively) than TATR, wherein they contributed 34.41% and 33.33% each (Fig. 4). The dholes did show significant selection of age-sex classes of chital (χ2 = 17.22, df = 2, *P* < 0.001) and sambar (χ2 = 17.44, df = 2, *P* < 0.001). In TATR, dholes harvested the fawns significantly more for both chital (χ2 = 13.30, df = 2, *P* < 0.001) and sambar (χ2 = 16.94, df = 2, *P* < 0.001).

**Figure 5:**
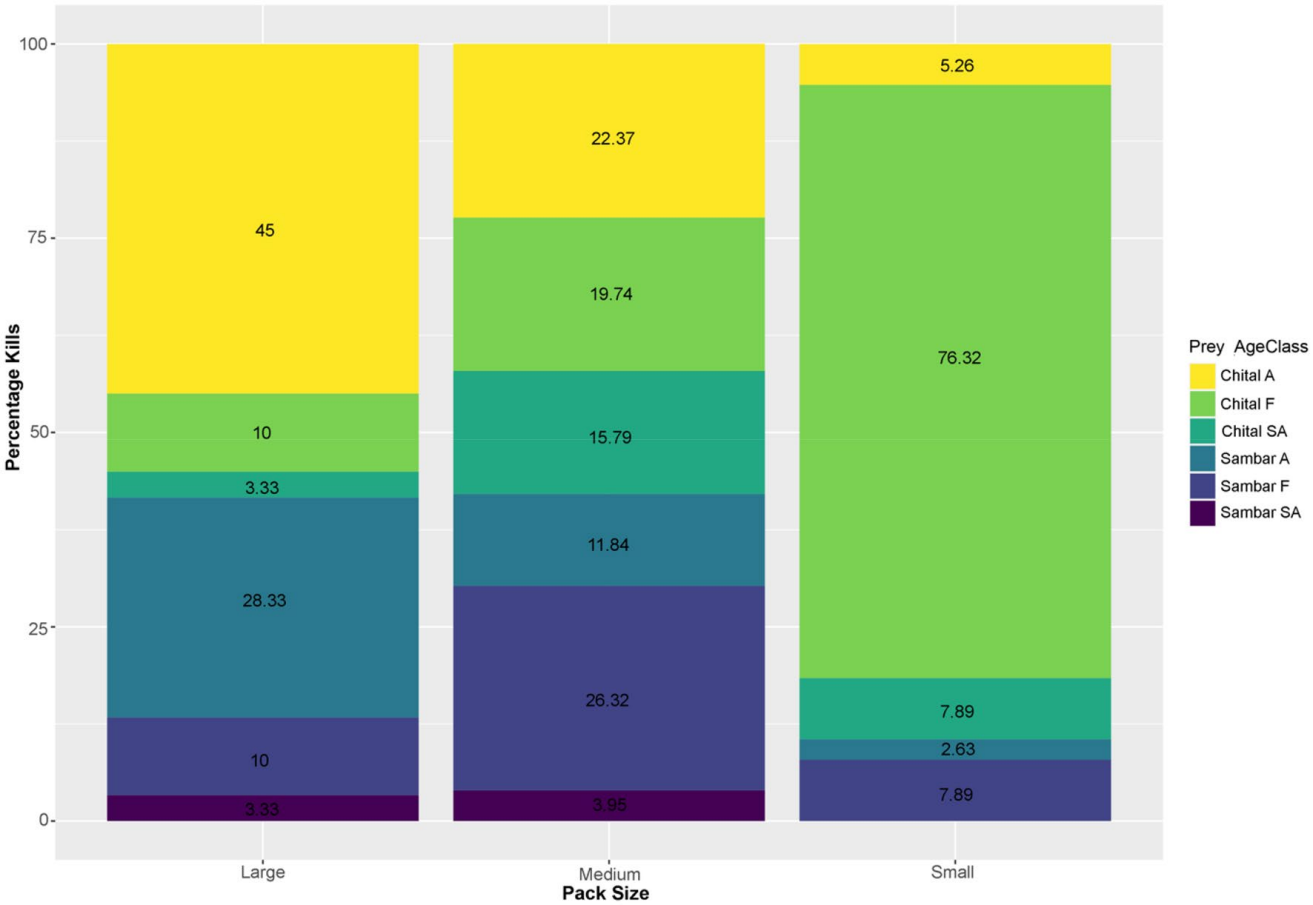
Relative proportion of principal prey with age classes (A—Adults, F—Fawns, SA—Sub Adults) harvested by dholes based on 3 categories of pack sizes (Large, Medium and Small) in TATR and NNTR from observed kills. Figure generated using the package “ggplot2” in R studio R Core Team (2019). R: A language and environment for statistical computing. R Foundation for Statistical Computing, Vienna, Austria. (https://www.R-project.org/).

Based on the 3 categories of pack sizes, we demonstrate that the kills (n=60) made by large packs consisted more adults of chital (45%) and sambar (28.33%) relative to other age classes. In contrast, the kills (n=38) made by the smaller packs consisted chital fawns (76.32%) more than other age classes (Fig. 5). No apparent preference for medium-sized packs, wherein prey was harvested from all size classes almost equally (n = 76).

## Discussion

Our study presents insights into the diet of the dholes in Central India by incorporating conventional diet analysis with molecular tools for confirming the identity of scats and radio-telemetry observations from the field to assess prey age-class. Our study also differs from previous dhole diet studies in terms of diet composition analysis in that, we chose to explore the exponential biomass models over linear ones and did not rely solely on the frequency of occurrence of prey^4,30^. The sample size was sufficient for 4 study sites (TATR, NNTR, STR, UKWLS). However, the sample size for the 2 sites (MTR, PTR) was insufficient for robust statistical analysis, and hence the results for these are to be considered preliminary trends.

The results from scat analysis establish that sambar and chital are the two principal prey species of dholes in the central Indian State of Maharashtra, comprising 80.16% of the biomass consumed and 53.11% of relative numbers of prey harvested. This finding is consistent with previous studies from Madhya Pradesh, another Central Indian State with tropical dry to moist deciduous forests^15,31,32^. The dhole diet also comprised chausinga (3.44%) in the landscape. Previously, another study from Nagarhole National Park in the Western Ghats reported 2% remains of chausinga in the dhole diet^33^. At the landscape scale, dholes preferred the small antelope occurring naturally in low-densities across its distribution range^34,35^ over other prey species after sambar and chital (Fig. 1). Dholes strongly avoided large, challenging prey like the gaur, nilgai and wild pigs (Fig. 1), however, these prey species were selectively preyed upon in some PAs. Previously as well, there have been records of dholes hunting calves of large prey like gaur and banteng^36^.

Based on the scat analysis, overall, dholes depended predominantly on large-sized prey like sambar (61.08%) and chital (19.08%), followed by gaur (4.02%) and nilgai (4.68%). Medium-sized prey like wild-pigs, barking deer, chausinga and langur comprised 10.17% of the biomass, and small-sized prey like the black-naped hare and rodents comprised 0.82% of the biomass of the dhole diet. The results suggest that medium to large-sized prey dominated the diet of dholes in the tropical dry and moist deciduous forests of Central India. The findings appear to converge with earlier studies that highlight the importance of wild ungulates, particularly sambar and chital. The exponential biomass model corrected for the bias of over-representation of smaller prey like rodents and hare (Supplementary 4). However, since biomass models are not yet based on feeding trials conducted specifically for dholes, we suggest it would be prudent to develop specific biomass equations for this species. We also found most scats with vegetative matter, mostly bamboo leaves or other grasses. Vegetation matter has been recorded previously in dhole diet^14,26,37,38^ and may probably aid in digestion or excretion of indigestible food items^39^. On one occasion, we observed a dhole feeding on a snake run over by a vehicle that we could not identify as the animal consumed it. A snake was recently recorded from a dhole scat^40^, but such instances are likely to be stray and not a part of the regular dhole diet.

On a PA scale, we found differential local densities of prey^41–47^, co-predator densities, and varying levels of human disturbance may lead to subtle variations in the diet pattern of dholes. For instance, we found sambar, wild pig and gaur cumulatively comprising 78.02% of the dhole diet in STR, whereas the chital contributed only 2.62% of the prey biomass. The rugged landscape of STR has naturally low densities of the gregarious, grassland species like the chital. In TATR, we also documented events of kleptoparasitism by larger predators (n = 7) wherein tigers stole dhole kills, or dholes took kills from leopards. The high competition over dietary resources within the carnivore guild may explain the lack of specialist scavengers such as hyenas, vultures, and jackals in the long-term monitoring at TATR. The occasional scavenging of tiger kills, and improper disposal of cattle carcasses may justify cattle remains in the dhole scats. Dholes have been known to scavenge kills of larger co-predators in high tiger density PAs across the landscape^15^. We did not observe or find any forest department records of livestock depredation incidents by dholes across our landscape, suggesting that the 1.57% representation of cattle in our data could be from scavenging events, suggesting that the depredation of livestock by dholes is extremely low. Our findings provide an opportunity for investigating such interactions in future.

The UKWLS, a small sanctuary with high human pressure, exhibited the widest niche breadth among the PAs in the landscape. Nilgai, a prey species associated with high human disturbance^31,48^, comprised 22.50% of the biomass of dhole diet in this PA, almost equal to that of sambar (See Supplementary Table S3). This could be attributed to lower densities of other prey species at UKWLS. Amongst the PAs, smaller sized prey consisted of 23.86% of the biomass in UKWLS. The finding could be attributed to a combination of factors such as high co-predator densities, the small size of PA, lack of high densities of alternative prey or individual choice. In TATR and NNTR, smaller sized species merely comprised 6.59% and 5.67% of the biomass in the dhole diet. The diet pattern of dholes in UKWLS concurs with a study on a population of African wild dogs that are known to subsist on smaller-sized prey in disturbed areas^49^.

Unlike the scat data, kills of chital had a higher representation (59.16%) than sambar (31.94%) in the kills made by dholes. This pattern has been observed from dhole diet studies based on observed kills^15^ and could be attributed to greater probabilities of encountering the hunts and remains of chital in open areas and grasslands. Sambar favour dense cover making it challenging to observe sambar hunts and detect kills. Dholes hunt on the move, eat hastily and are not known to return to their kills. Due to such hunting habits, dholes never form ‘location clusters', attributed to kills like most predators^50–54^. Furthermore, while recording kills, small prey may be missed. Regardless of these limitations, observations of dhole kills revealed much about the age-sex classes of principal prey species in our study, as discussed further.

Our data suggest that in NNTR, adults of chital and sambar contributed more to the kill composition of dholes, whereas in TATR, fawns contributed more to the kills in the dhole diet. The selection of fawns in TATR may be attributed to the presence of a high density of tigers, the dominant predator, compared to the low density in NNTR at the time of our study (0.46/100 km^2^)^55^. Fawns are easier to subdue and are likely to result in reduced duration of kill retention, thereby limiting unwanted detection by the other predators, but may still allow for energetic demands to be fulfilled, as reported in the case of the African carnivore guild^56^. Parker et al*.*^57^ reported a similar pattern wherein cheetahs caught smaller prey in the presence of lions at particular study sites. Additionally, this pattern could be a manifestation of larger mean pack sizes in NNTR^55^ compared to TATR. On categorising overall data from both study sites based on pack size, we found support for this argument wherein kills from larger pack sizes comprised 73.33% of adult prey. In contrast, smaller packs consisted of 84.21% of fawns. Although previous studies from Bandipur National Park and Pench Tiger Reserve based on observed kills have demonstrated the selection of fawns in dhole diet, both study sites had large mean pack sizes^15,58^. Reduced intra-guild competition due to extremely low densities of a dominant predator like the tiger, and not just large pack sizes, may enable dholes to hunt large prey in NNTR.

Studies on dietary preferences are vital for carnivore ecology, conservation and management. Since the loss of prey base is one of the major threats to dhole populations across their distribution^13^, studies on the feeding ecology of these endangered canids are crucial. Being the first diet study from a region with one of the highest dhole occupancies within India, our study fills an important gap in the dhole diet literature from the country. Our findings concur with other studies that state that wild ungulates are a vital resource for the survival of dholes and highlight that domestic prey rarely contributes to the dhole diet in the region. We caution that common resources may lead to intense competition in a high-density multi-predator system. From a management perspective, maintaining relatively high densities of preferred ungulate prey species might be especially important for conserving a hyper carnivorous canid species like the dhole amidst other co-predators. We suggest complimenting scat analyses with kills data whenever possible, especially useful while comparing carnivore diets^59,60^. In the case of dholes, GPS clusters are not representative of kill sites as they hunt on the go and consume their prey hastily. However, other than kill clusters, radiotelemetry can aid in assessing critical behaviours like co-predator interactions, energy budgets, kill rates and estimating hunting success^54^. Calibrating sampling intervals, fix rates and using sophisticated activity sensors can influence the probability of locating kill sites. Researchers and managers can recognise these limitations and opportunities to select methods that best fit their objectives, budget, and fieldwork logistics.

## Methodology

### Study area

We conducted the study across the CI landscape in Maharashtra State, India. The samples were collected from 6 protected areas– Tadoba Andhari Tiger Reserve (TATR—1727 km^2^), Pench Tiger Reserve (PTR—257.3 km^2^), Melghat Tiger Reserve (MTR—1500.49 km^2^), Nagzira-Nawegaon Tiger Reserve (NNTR—152.8 km^2^), Sahyadri Tiger Reserve (STR—1166 km^2^) and Umred Karhandla Wildlife Sanctuary (UKWLS—189 km_2_). The vegetation in the study areas is primarily of dry and moist deciduous forest type^61^. These forests support a diverse assemblage of co-predators and prey species, such as the tiger (*Panthera tigris*), leopard (*Panthera pardus*), sloth bear (*Melarsus ursinus*), wolf (*Canis lupus*), and other small carnivores (canids, felids, viverrids and herpestids). Large prey species like sambar (*Cervus unicolor* Kerr), chital (*Axis axis*), gaur (*Bos gaurus*), nilgai (*Boselaphus tragocamelus*), wild pig (*Sus scrofa*), barking deer (*Muntiacus muntjak*), four-horned antelope or chausinga (*Tetracerus quadricornis*), hanuman langur (*Semnopithecus entellus*) and bonnet macaque (*Macaca radiata*) also occur. Additionally, several smaller prey species such as mouse deer (*Moschiola indica*), black-naped hare (*Lepus nigricollis*), and rodents are also found in the landscape. These protected areas are known habitats of dholes in the mosaic landscape of dense forests and intensive human use areas.

### Field methods

The study areas have an extensive network of forest roads that facilitate the collection of scats deposited by the dholes at latrine sites and junctions of trails/roads. We collected 1,407 dhole scats and used 861 samples to study the dhole diet profile. We did not use highly degraded scats that had no identifiable undigested prey remains for further analysis. Following Thinley et al.^62^, we randomly collected one scat from latrines containing 2–4scats, two scats from latrines containing 5–8 scats, and three scats from latrines containing > 8 scats in order to avoid biasing our results toward a few large latrines and to ensure different feeding events were more equally sampled. We discarded unused scats off the trails. Dhole scats were easily identified in the field and distinguished from all other predator scats by the presence of dhole tracks, the location of scat deposition (at trail/road junctions) and the characteristic collective deposition of scats by a pack. The scat identity was further confirmed by its distinct odour and appearance. We also corroborated our field identification skills by using molecular tools. To ensure the dataset was robust, we genetically verified a subset of freshly collected scats (n = 623) correctly assigned for 100% of the samples^63,64^. Therefore, there was a minimal potential for misidentification of scats of dholes from other carnivores, limiting bias in our results. We also tracked radio-collared individuals from known packs (n = 5) to collect scats and record opportunistic direct kill observations during periods of hunting activity (dawn and dusk) at TATR and monitored the kills made by known packs from NNTR (n = 5). From the 2 sites, we analysed dhole kills to assess the dietary preferences of dholes. We could follow packs on foot if required to locate kills, so our observations were not limited to main roads, and sampling was unbiased except perhaps for very small prey (See Supplementary Fig. S5). The study sites were monitored across seasons for 3 years (2018, 2019, 2020) by trained research teams of long term monitoring projects. The latrines of the same pack were not sampled for 7 days after a scat was collected to minimize the chance of sampling multiple scats from the same feeding event.

### Lab methods

Identification of prey hair found in scats is a common, non-invasive approach used to study diet composition of predators^65,66^ and has been followed globally for dholes as well (See Supplementary Table S1). For a reliable estimate of the diet preferences of dholes, standard prescribed protocols were adopted^67^. Observation slides of randomly picked hair –a minimum of 20 prey hairs per scat, were washed in ethanol and a xylene bath and examined under a compound microscope^68^. We identified the prey species by comparing medullary characteristics of hair with known standard references and slides at the Wildlife Institute of India, Dehradun and slides that the team prepared from carcasses in the field. Other miscellaneous indigestible prey remains (claws, bones, or teeth) recovered from the processed scat samples also contributed to identifying prey species. We also recorded any undigested plant material found in the scats (mostly bamboo leaves). We corroborated the identity of a subset of scats (n = 623) at the species level using genetic analysis^63,64^. Scats that were highly degraded or had too few identifiable prey remains were removed from the analysis.

### Diet analyses

We calculated the frequency of occurrence^69^ and the biomass of prey consumed^1^ to avoid drawing erroneous conclusions based on the recommendations of Klare et al.^4^. The frequency of occurrence may over-represent smaller prey as they tend to produce relatively more scats per kilogram of meat consumed than larger prey. Relative biomass models aid in overcoming this bias by accounting for prey species body mass^70,71^. Previous studies opted for a correction factor developed for gray wolves^1^ *Canis lupus* with modifications^72^ to estimate the relative importance of the prey species. Wachter et al.^30^ refined the linear regression biomass model wherein an exponential model was proposed accounting for the maximum consumable biomass for a species. Models where the graph of consumable biomass reaches an asymptote, are more accurate and ecologically meaningful^73,74^. Since the biomass consumed accounts for differential digestibility of food items^4^ and is considered the most relevant parameter ecologically, we used the non-linear model for our analyses:

$${\text{Y }} = { 1}.{382 }\left( {{1} - {\text{ exp}}\left( { - 0.0{\text{21X}}} \right)} \right)$$

where Y is the biomass of prey consumed (kg) to produce a single field collectable scat and X is the mean bodyweight of the prey species (kg). The mean live weight of potential prey species hunted by dholes used in our analyses were based on previously published literature on dhole dietary preferences^33,34^. Using the species-specific correction factor Y, we computed the frequency of occurrence (A), relative biomass (D) and the relative number of prey species consumed (E) (expressed as percent):

$${\text{D }} = \, \left( {{\text{A }}*{\text{ Y}}} \right) \, / \, \Sigma \, \left( {{\text{A }}*{\text{ Y}}} \right) \, *{ 1}00$$

$${\text{E }} = \, \left( {{\text{D }}/{\text{ X}}} \right) \, / \, \Sigma \, \left( {{\text{D }}/{\text{ X}}} \right) \, *{ 1}00$$

We used the Jacobs' index^75^ to compute prey electivity for all the study sites using the formula:

$${\text{Jacobs' Index }}\left( {\text{D}} \right) \, = {\text{r }}{-}{\text{ p}}/{\text{ r }} + {\text{ p }}{-}{\text{ 2pr}}$$

where r is the proportion of total kills of each prey species, and *p* is the proportion of the total availability of the prey species. The resulting values of the index range from + 1 to − 1, indicating maximum preference and maximum avoidance, respectively. Prey densities, availability and utilisation of prey species across study sites was obtained from long term research in the area^41–47,76^ and the Government of India livestock census^77^. We did not estimate the relative biomass consumed and selectivity for rodents, birds, and vegetative matter because of uncertainty about their weights and densities.

We evaluated the niche breadth using the Levins’ standardized niche breadth index (B)^78^:

$$B_{s} = \frac{{\frac{1}{{\sum p_{i,s}^{2} }} - 1}}{n - 1},$$

where s is the focal species, p_i,s_ is the proportion of individuals of species s found on niche i, and n is the number of niches available. This index is a modification of the basic Levins’ niche breadth index, with the advantage of scores scaled between 0 and 1 (wherein 0 is a diet specialist and 1 diet generalist).

### Dietary patterns based on kills in the wild

We recorded the data on dhole kills from two study sites with intensive tracking of packs- TATR and NNTR. Since scat analysis alone cannot inform us about the age classes of prey taken, data on kills becomes essential in filling this gap. Dhole packs were radio-collared in TATR (n = 4) and another pack with 2 individuals with distinct marks were monitored across seasons over 3 years. Radio-collared dholes were fitted with GPS radio collars (Vectronics, Berlin, Germany) with a UHF (ultra-high frequency) ground download system^79^. Packs in Nagzira (n = 5) were monitored based on distinct scars or markings. We reported the relative proportion of all the prey species harvested based on pack size categories—small, medium and large. The small pack sizes consisted of 2–4 individuals; medium pack sizes consisted of 5–8 individuals; and large packs had > 8 individuals. Further, following Acharya^15^, we compared the age-class distribution of the two major prey species—sambar and chital in the kill data, to check if dholes were selecting for a particular age class of each prey. We carried out statistical comparisons using a Chi-square test in conjunction with Bonferroni's simultaneous confidence intervals^80^. We used the χ2 goodness-of-fit test to indicate whether there was significant selectivity of age-sex class, and the 95% confidence intervals for observed proportions were estimated. We compared observed proportions with their corresponding expected proportions using z-tests to evaluate under- or over-representation of groups if the chi-square test was significant. We set a 5% level of significance to evaluate the significance of association, i.e. the *p*-value was considered significant if it was less than 0.05. We used Microsoft Excel 2019, the R software (v.1.3.959) and ArcGIS Pro version 3.0 for all statistical analyses and graphical representations.

### Ethics declaration

Dhole capture permit (MFD-SPP-12/05.11.2016) for the radio-collared dholes in the study.

## Supplementary Information

Supplementary Information.

## Data Availability

All data generated or analysed during this study are included in this article or as Supplementary Information files. Any additional information are available from the corresponding author on reasonable request.
