# Supplementary material for: Feeding ecology of the endangered Asiatic wild dogs (Cuon alpinus) across tropical forests of the Central Indian Landscape
Source: Sci Rep. 2022 Aug 18;12:14029. doi: 10.1038/s41598-022-17906-5 (PMC9388674; doi:10.1038/s41598-022-17906-5)
Supplement: Supplementary file 1 — Supplementary Information. [file 41598_2022_17906_MOESM1_ESM.pdf]

Supplementary S1

Table: Details of 36 diet assessments of dhole diet preferences (1975 to 2021) across the 3 landscapes in India and across Asia. Mode of study refers to the methodology followed to estimate the prey species. Biomass Model refers to the linear vs exponential equation used for estimating prey biomass in dhole diet (NA- not applicable as these studies may not have estimated the Biomass).

Sr. No.	Author	Study site	Landscape in India	Mode	Biomass Models
1	Fox and Johnsingh 1975	Mudumalai Tiger Reserve, India	Western Ghats, Tamil Nadu	Scat Analysis- Prey Remains Identification	NA
2	Cohen et al. 1978	Mudumalai Tiger Reserve, India	Western Ghats, Tamil Nadu	Scat Analysis- Prey Remains Identification	NA
3	Barnett et al. 1980	Mudumalai Tiger Reserve, India	Western Ghats, Tamil Nadu	Scat Analysis- Prey Remains Identification	NA
4	Johnsingh 1983	Bandipur National Park, India	Western Ghats, Karnataka	Scat Analysis- Prey Remains Identification	NA
5	Rice 1986	Eravikulam National Park, India	Western Ghats, Kerala	Scat Analysis- Prey Remains Identification	NA
6	Johnsingh 1992	Bandipur National Park, India	Western Ghats, Karnataka	Observed Kills, Scat Analysis- Prey Remains Identification	NA
7	Karanth and Sunquist 1995	Nagarhole National Park, India	Western Ghats, Karnataka	Observed Kills, Scat Analysis- Prey Remains Identification	Linear
8	Venkatraman et al. 1995	Mudumalai Tiger Reserve, India	Western Ghats, Tamil Nadu	Observed Kills, Scat Analysis- Prey Remains Identification	Linear
9	Acharya 2007	Pench Tiger Reserve, India	**Central India,**	Observed Kills, Scat Analysis- Prey	Linear

			**Madhya Pradesh**	Remains Identification	
10	Andheria et al. 2007	Bandipur National Park, India	Western Ghats, Karnataka	Scat Analysis- Prey Remains Identification	Linear
11	Joseph et al. 2007	Parambikulam Wildlife Sanctuary, India	Western Ghats, Kerala	Scat Analysis- Prey Remains Identification	Linear
12	Edgaonkar 2008	Satpuda Tiger Reserve, India	**Central India, Madhya Pradesh**	Scat Analysis- Prey Remains Identification	Linear
13	Borah et al., 2009	Satpuda Tiger Reserve, India	**Central India, Madhya Pradesh**	Scat Analysis- Prey Remains Identification	Linear
14	Kumaraguru et al. 2010	Anamalai Tiger Reserve, India	Western Ghats, Tamil Nadu	Scat Analysis- Prey Remains Identification	Linear
15	Majumder et al. 2011	Pench Tiger Reserve, MP, India	**Central India, Madhya Pradesh**	Scat Analysis- Prey Remains Identification	Linear
16	Ramesh et al. 2012	Mudumalai Tiger Reserve, India	Western Ghats, Tamil Nadu	Scat Analysis- Prey Remains Identification	Linear
17	Selvan et al. 2013a	Review, India			NA
18	Selvan et al. 2013b	Pakke Tiger Reserve, India	North East, Arunachal Pradesh	Scat Analysis- Prey Remains Identification	NA
19	Bashir et al. 2014	Khangchendzonga Bioshpere Reserve, India	North East, Sikkim	Scat Analysis- Prey Remains Identification	Linear
20	Hayward et al., 2014	Review, India			NA
21	Lyngdoh et al. 2014	Arunachal Pradesh	North East, Arunachal Pradesh	Scat Analysis- Prey Remains Identification	Linear
22	Dar and Khan 2016	Silent Valley National Park, India	Western Ghats, Kerala	Scat Analysis- Prey Remains Identification	Linear
23	Srivathsa et al., 2020	Review, India			**Non-linear**

24	George et al., 2021	South Wayanad Forest Division	Western Ghats, Kerala	Scat Analysis- Prey Remains Identification	Linear
Other dhole range countries					
26	Aryal et al., 2015	Dhorpatan Hunting Reserve (DHR), Nepal		Scat Analysis- Prey Remains Identification	Linear
27	Wang & MacDonald, 2009	Jigme Singye, Bhutan		Scat Analysis- Prey Remains Identification	Linear
28	Thinley et al., 2011	Jigme Dorji, Bhutan		Scat Analysis- Prey Remains Identification	Linear
29	Grassman et al., 2005	Phu Khieo, Thailand		Scat Analysis- Prey Remains Identification	NA
30	Kawanishi & Sunquist, 2008	Taman Negara, Malaysia		Genetic ID, Scat Analysis- Prey Remains Identification	Linear
31	Kamler et al., 2012	Nam Et-Phou Louey, Laos		Genetic ID, Scat Analysis- Prey Remains Identification	Linear
32	Nurvianto et al., 2016	Baluran, Indonesia		Scat Analysis- Prey Remains Identification	Linear
33	Khoewsree et al., 2020	Khao Yai National Park, Thaliand		Scat Analysis- Prey Remains Identification	NA
34	Charaspet et al., 2020	Khao Yai National Park, Salak Pra, Huai Kha Khaeng Wildlife Sanctuaries, Thailand		Scat Analysis- Prey Remains Identification	Linear
35	Kamler et al., 2020	Nam Et-Phou Louey National Protected Area, Laos; Srepok Wildlife Sanctuary, Cambodia		Scat Analysis- Prey Remains Identification	Linear
36	Steinmetz et al,. 2021	Kuiburi National Park, Thailand		Scat Analysis- Prey Remains Identification	Linear

Supplementary S2

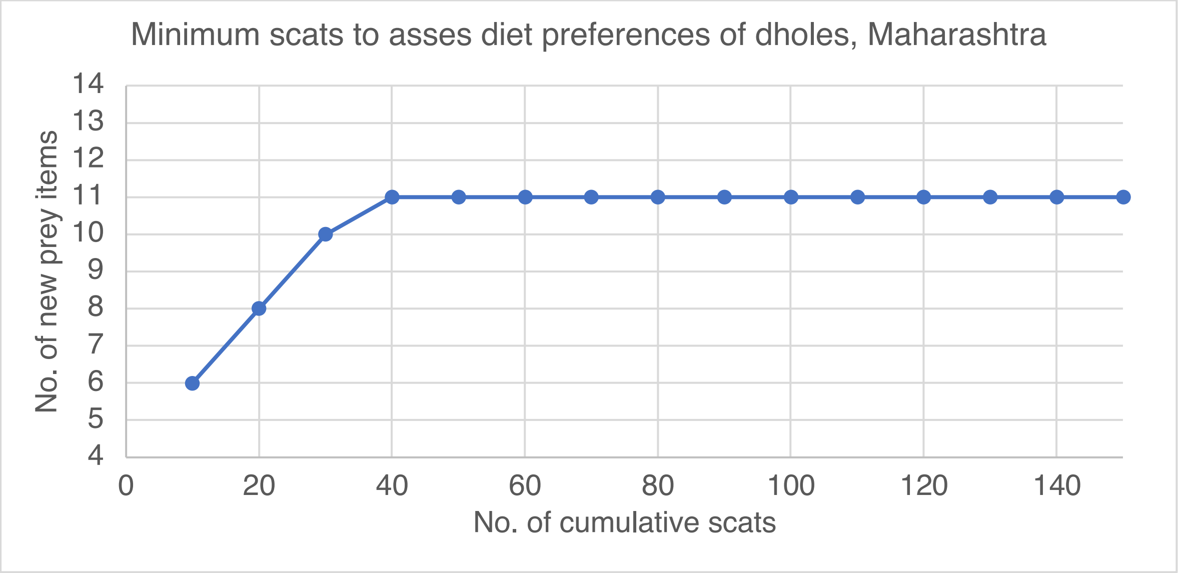

Fig 1.: Calculation of sample adequacy of dhole scats in Maharashtra, India with an asymptote at 40 scats.

Supplementary S3

Table: Frequency of occurrence (A), relative biomass consumed (D) and relative number of prey individuals consumed (E) by dhole packs at individual study sites. TATR – Tadoba Andhari Tiger Reserve; NNTR – Nawegaon-Nagzira Tiger Reserve; STR – Sahyadri Tiger Reserve; UKWLS – Umred Karhandla Wildlife Sanctuary; MTR – Melghat Tiger Reserve; PTR – Pench Tiger Reserve.

	TATR			NNTR			STR			UKWLS			MTR			PTR		
**Prey**	**A**	**D**	**E**	**A**	**D**	**E**	**A**	**D**	**E**	**A**	**D**	**E**	**A**	**D**	**E**	**A**	**D**	**E**
**Chital**	19.11	21.25	17.95	15.70	19.62	13.83	1.82	2.62	1.50	8.55	18.19	6.28	7.69	10.10	6.61	26.32	30.28	24.22
**Sambar**	53.78	63.56	47.64	47.09	62.57	39.12	39.09	59.79	30.39	10.26	23.20	7.11	53.85	75.11	43.62	52.63	64.37	45.68
**Barking Deer**	3.85	2.14	4.98	1.16	0.73	1.41	4.55	3.28	5.16	11.11	11.84	11.25	0.00	0.00	0.00	0.00	0.00	0.00
**Black naped hare**	2.62	0.26	4.02	13.37	1.49	19.25	6.36	0.82	8.57	22.22	4.22	26.70	7.69	0.90	10.80	10.53	1.08	15.84
**Gaur**	3.24	4.17	2.58	0.58	0.84	0.43	5.45	9.09	3.82	2.56	6.32	1.60	0.00	0.00	0.00	0.00	0.00	0.00
**Nilgai**	2.77	3.47	2.30	4.07	5.72	3.17	1.82	2.94	1.32	9.40	22.50	6.11	0.00	0.00	0.00	0.00	0.00	0.00
**Rodent**	3.24	0.03	5.11	3.49	0.03	5.17	6.36	0.07	8.82	24.79	0.40	30.65	0.00	0.00	0.00	0.00	0.00	0.00
**Chausinga**	5.70	3.05	7.45	3.49	2.09	4.27	10.91	7.54	12.51	6.84	6.99	6.99	0.00	0.00	0.00	5.26	2.91	6.74
**Langur**	4.47	1.12	6.52	4.65	1.31	6.36	14.55	4.73	18.62	0.85	0.41	0.98	23.08	6.84	30.78	5.26	1.37	7.52
**Wild Pig**	1.23	0.96	1.44	6.40	5.58	6.98	9.09	9.14	9.29	2.56	3.81	2.34	7.69	7.05	8.19	0.00	0.00	0.00
**Cattle**	0.00	0.00	0.00	0.00	0.00	0.00	0.00	0.00	0.00	0.85	2.11	0.00	0.00	0.00	0.00	0.00	0.00	0.00

Supplementary S4

Table: Comparison of biomass models D_L vs. D_E (linear vs. exponential equations) to demonstrate the difference between prey biomass in the dhole diet based on scat sampling across the Central Indian Landscape of Maharashtra, India. The exponential model (D_E) corrects for bias of over representation of smaller prey.

Prey	Prey Weight (X)	Biomass Linear (D_L) (Floyd et al., 1978)	Biomass Exponential (D_E) (Wachter et al., 2012)
Rodent	0.25	1.68	0.05
Black naped Hare	3	2.40	0.77
Langur	8	2.24	1.49
Chausinga	19	3.43	3.44
Barking Deer	20	2.61	2.66
Wild Pig	31	2.33	2.58
Chital	55	17.78	19.08
Sambar	62	58.62	61.08
Nilgai	70	4.67	4.68
Gaur	75	4.11	4.02
Cattle	75	0.14	0.13

Floyd, T. J., *et al.* Relating Wolf Scat Content to Prey Consumed. *Journal of Wildlife Management* **42:**528-532 (1978).

Wachter, B. *et al.* An Advanced Method to Assess the Diet of Free-Ranging Large Carnivores Based on Scats. *PLOS ONE* **7**, e38066 (2012).

Supplementary S5

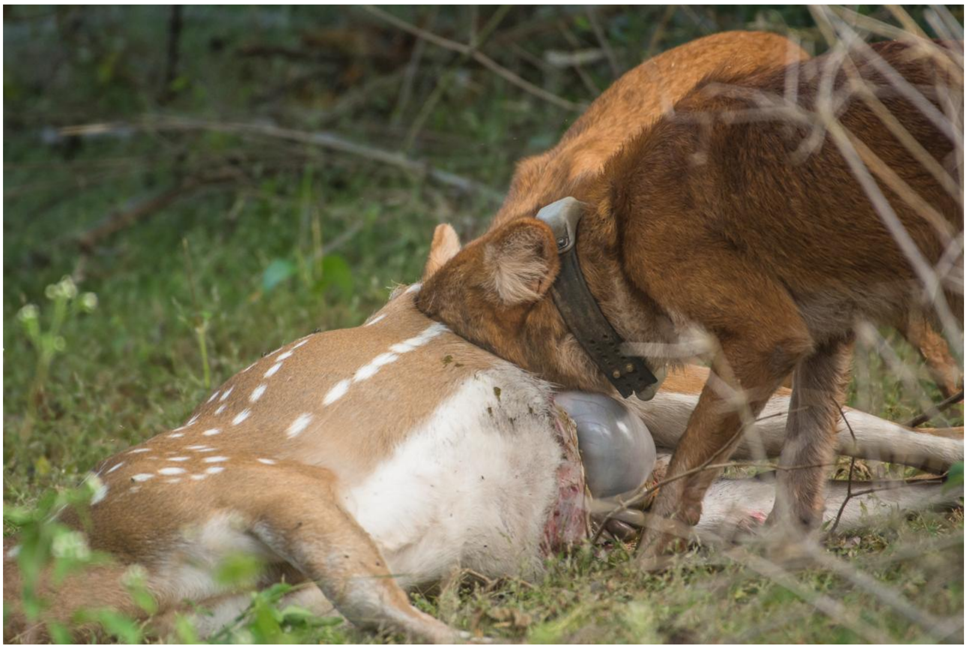

a)

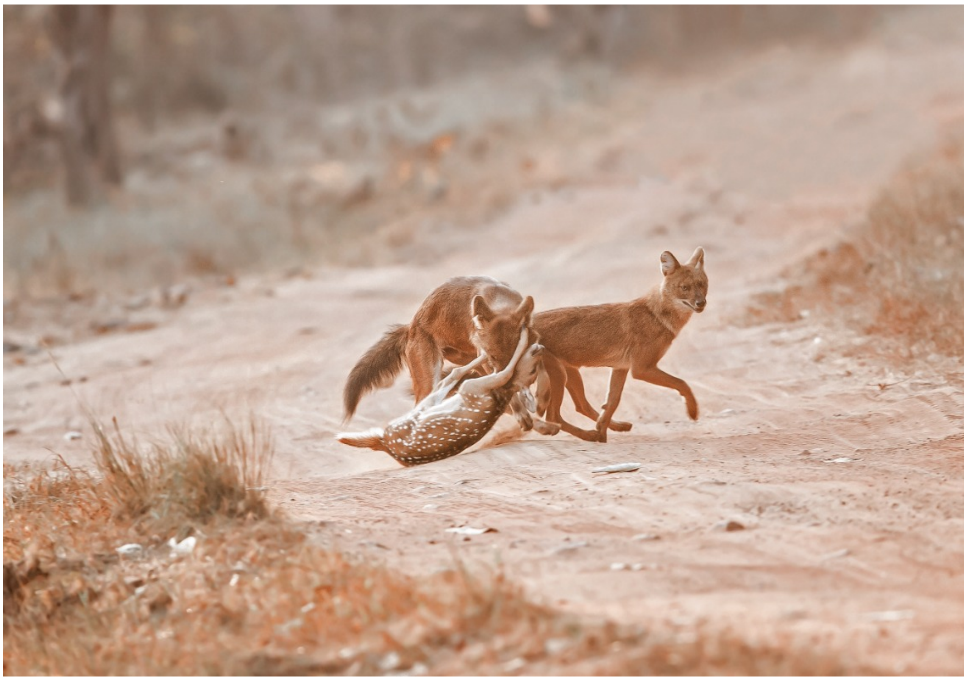

b)

Fig. a) A radio-collared dhole feeding on a chital carcass with the pack.; b) A dhole pack killing a chital fawn.
